# Assessment of Cheese Contamination and Its Contribution to Aflatoxin M1 Intake in the Spanish Population

**DOI:** 10.3390/foods15040720

**Published:** 2026-02-15

**Authors:** Susana Lorán, Marta Herrera, Agustín Ariño, Teresa Juan

**Affiliations:** 1 Facultad de Veterinaria, Instituto Agroalimentario de Aragón-IA2, Universidad de Zaragoza-CITA, 50013 Zaragoza, Spain; herremar@unizar.es (M.H.); aarino@unizar.es (A.A.); tjuan@cita-aragon.es (T.J.); 2 Centro de Investigación y Tecnología Agroalimentaria de Aragón (CITA), Avda. Montañana 930, 50059 Zaragoza, Spain

**Keywords:** aflatoxin M1, cheese, occurrence, exposure

## Abstract

Global concern surrounds the contamination of dairy products with aflatoxin M1 (AFM1), a mycotoxin found in the milk of ruminants fed with aflatoxin B1-contaminated feed. Among dairy products, cheese is of the foods with the highest concentration of AFM1 mycotoxin, although the reported levels vary widely. This study analyzed AFM1 levels in 100 commercial cheeses produced and marketed in Spain. AFM1 was detected in 51% of the samples, with concentrations ranging from 8.1 to 470.7 ng/kg. The milk type and animal species significantly influenced the contamination levels with a higher prevalence and mean contamination rates in cheeses made from cow’s milk (67.6% and 43 ng/kg) and pasteurized milk (60.7% and 33 ng/kg). The degree of ripening of the cheese did not significantly influence the contamination of the products, although the occurrence and average levels observed in fresh cheeses (63.0% and 53 ng/kg) were higher than those observed in mature (48.0% and 13 ng/kg) and semi-mature cheeses (43.5% and 11 ng/kg). There were no significant differences in the contamination levels between artisanal (56.6% and 33 ng/kg) and industrial (44.7% and 12 ng/kg) cheeses. The dietary exposure estimates for average consumers were low across all age groups: 0.004 ng/kg bw/day (adults), 0.007 ng/kg bw/day (adolescents), 0.025 ng/kg bw/day (children), and 0.081 ng/kg bw/day (toddlers). Consequently, the Margin of Exposure (MOE) values exceeded 10,000, indicating a low public health concern, except for toddlers in the 95th percentile of consumption.

## 1. Introduction

Aflatoxins, secondary metabolites produced by toxigenic fungi, are considered among the most significant mycotoxins. They are synthesized by various species of *Aspergillus*, primarily *A. flavus* and *A. parasiticus*, as well as *A. nomius* [1]. These fungi may contaminate crops and feedstuffs during growth, transport, and storage, although contamination by *Aspergillus* species is most frequently associated with post-harvest stages.

From a food safety perspective, the most important aflatoxins are aflatoxin B1 (AFB1), B2 (AFB2), G1 (AFG1), G2 (AFG2), and M1 (AFM1).

AFM1 is a monohydroxylated derivative of AFB1 formed in the livers of animals that ingest feed contaminated with this mycotoxin, a process involving enzymes associated with cytochrome P450. The resulting AFM1 is excreted by lactating animals through the mammary glands into milk [2,3]. The relationship between the amount of AFB1 ingested and the amount of AFM1 excreted is influenced by factors such as the species, lactation stage, milk yield, and duration of exposure [3]. Although the conversion rate is generally low, ranging from 0.3 to 6.2% in cows, AFM1 has been detected in milk from various species and sources. Because aflatoxins can contaminate feedstuffs such as grains, oilseeds, forages, and concentrates, the quantification of AFB1 in feed [4,5,6] and the subsequent contamination of dairy products [7,8] have been extensively documented.

This contamination is a global concern, as long-term exposure can affect immunological and liver health, as well as kidney function, which can have serious implications for human and animal health. It is well established that AFB1, the aflatoxin for which the most toxicological information is available, has a carcinogenic potency approximately one order of magnitude higher than that of AFM1. Still, the International Agency for Research on Cancer (IARC) has classified AFM1 in Group 1 due to its strong evidence of carcinogenicity because of a genotoxic mode of action [9]. Therefore, exposure to AFM1 remains a cause for concern. As there is no safe exposure threshold for AFM1, no tolerable daily intake (TDI) values have been established. However, risk evaluation is the most effective method for assessing the potential links between food hazards and risks to human health. Therefore, the recommended strategy is to minimize exposure using the ALARA (as low as reasonably achievable) principle.

The necessity for updated assessments of AFM1 in dairy products is further underscored by the projected impact of climate change on European agriculture. Predictive models suggest that temperature increases will significantly elevate the risk of *Aspergillus flavus* proliferation and aflatoxin B1 contamination in key feedstocks such as corn and corn silage, particularly in the Mediterranean basin [10,11]. These studies predicted that a +2 °C climate scenario would significantly increase the risk of *Aspergillus flavus* and aflatoxin B1 contamination in maize across Southern Europe, including Spain and Italy. By 2050, approximately half of the European regions suitable for maize cultivation may face a medium-to-high probability of contamination, as rising temperatures and humidity promote the northward spread of mycotoxin-producing fungi. Rising temperatures and humid conditions are actively promoting the spread of mycotoxin-producing fungi into previously rare regions. In addition, recent full-chain modeling indicates these environmental shifts could increase the AFM1 levels in milk by up to 50% by 2030 [12]. Consequently, historical data, such as previous Spanish surveys reporting zero detection [2], may no longer accurately reflect the contemporary risk landscape.

Milk is an important component of the human diet across all age groups due to its high content of essential nutrients, including proteins, lipids, and calcium. When evaluating exposure to aflatoxin M1 (AFM1), milk is not the only dietary source. Owing to its high stability, AFM1 can persist in dairy products manufactured from contaminated milk. Among these products, cheese usually has the highest AFM1 concentration, probably due to its affinity for the hydrophilic portion of casein, which results in a higher concentration in the curd than in the whey [4]. While research consistently demonstrates elevated AFM1 concentrations in cheese compared with the raw milk used, the data remain heterogeneous; the contamination levels vary depending on the producing species, milk quality, cheese type, and cheesemaking process [3,13]. The high concentrations reported in meta-analyses suggest that cheese consumption may present a potential health risk [14].

In the EU, Commission Regulation (EU) 2023/915 [15] establishes a maximum level for AFM1 of 0.05 μg/kg in raw milk, heat-treated milk, and milk for the manufacture of dairy products. However, no maximum value is specified for dairy products themselves. Article 3 of the aforementioned regulation states that when no specific EU maximum levels have been set for dehydrated, diluted, or processed foods, the food business operator must provide and justify the processing factors. In contrast, some countries have established specific limits for cheese, such as Switzerland [16] and Iran [17] (250 ng/kg), Italy (450 ng/kg) [18], and Brazil (2500 ng/kg) [19].

While global meta-analyses have synthesized the broad risks of AFM1 in dairy, there is a lack of recent comprehensive data for Southern Europe, a region with high cheese diversity. In this context, the present study aimed to assess the occurrence of AFM1 in commercial cheeses marketed in Spain and to evaluate the contribution of cheese consumption to dietary exposure in different population groups.

## 2. Materials and Methods

### 2.1. Sampling Collection and Preparation

A total of 100 cheese samples, representing various species (cow, sheep, goat, and their mixtures) and maturity levels, were assessed for the presence of aflatoxin M1 (AFM1). All cheese samples were purchased in 2024 from retail stores and supermarkets located in the northeastern regions of Aragón and Cataluña. While the procurement was in these two regions, the samples originated from 12 different autonomous communities (see Supplementary Figure S1). This study design prioritized reflecting consumer exposure at the national market level over a strictly exhaustive regional survey.

To ensure a representative overview, a stratified sampling method was employed. The selection encompassed the most widely consumed categories in Spain, fresh, semi-cured, and cured, alongside the four primary milk sources. By sourcing from high-turnover supermarkets and artisanal shops, the study captured products from various regional and major nationwide brands. Although this dataset represents a contemporary snapshot, the inclusion of industrial and artisanal systems provides a comprehensive assessment of current national AFM1 exposure levels.

Once in the laboratory, samples from mature cheeses were grated using a manual grater with the smallest possible diameter (2 mm). Samples from unripened cheeses or with spreadable textures were homogenized using a fork and/or spoon. The prepared and coded samples were stored in sample containers with lids and frozen at −20 °C (±1 °C) until analysis.

### 2.2. Aflatoxin M1 Analysis

We used a methodology previously optimized in the laboratory that was based on the procedures described by Iha et al. [20] and the provisions of standard ISO 14501:2022 [21] and validated in accordance with the criteria of linearity, accuracy, precision, and sensitivity established in the Commission Implementing Regulation (EU) 2023/2782 [22].

The AFM1 analysis was conducted as follows: about 8 g of each homogenized sample was weighed in a conical tube followed by the addition of 2 g of sodium chloride (Chem-Lab, Zedelgem, Belgium), 22 mL of HPLC-grade methanol (Chem-Lab, Zedelgem, Belgium), and 13 mL of milli-Q water (Millipore, Bedford, MA, USA). This mixture was homogenized in a rotary shaker for 30 s, after which it was transferred to an orbital shaker at maximum speed for a further 30 min. The samples were then subjected to a centrifugation process at 4200 rpm for 30 min. After centrifugation, the residual fatty layer was separated, and 30 mL of the product was diluted with 60 mL of Milli-Q water. The homogenized mixture was then filtered through a glass microfiber filter (Whatman, Maidstone, UK) and collected in an amber glass vial until purification using immunoaffinity columns.

A volume of 60 mL of the filtrate was transferred into a syringe barrel attached to an immunoaffinity clean-up column (IAC Afla M1, VICAM, Watertown, MA, USA) at a flow rate of 1–2 drops per second. The column was rinsed with 20 mL of milli-Q water to eliminate any impurities. Next, 1.25 mL of acetonitrile/methanol (3:2 *v*/*v*) and 1.25 mL of milli-Q water were passed through the column to elute aflatoxin M1. The eluate was finally filtered using a 0.2 μm filter, placed into autosampler vials, and analyzed.

Identification and quantification of AFM1 in the purified extracts of the cheese samples was accomplished using a chromatographic Acquity UPLC H-Class system coupled with a 2475 multi-fluorescence detector and controlled using Empower 3 software (Waters Corp., Milford, MA, USA). Chromatographic separation was achieved on an Acquity UPLC HSS T3 column (150 mm × 2.1 mm × 1.8 mm, Waters Corp., Milford, MA, USA), using an isocratic mobile phase consisting of water/acetonitrile/methanol (68:24:8, *v*/*v*/*v*), acidified with formic acid to pH 2.0. Separation was achieved using a pump operating at a flow rate of 0.2 mL/min in isocratic mode. The samples and the column were maintained at temperatures of 5 and 35 °C, respectively. The injection volume was 15 µL, and the total duration of the process was 10 min. The retention time of AFM1 was approximately 6.25 min. The fluorescence detector wavelength was set at 360 nm for excitation and 440 nm for emission.

For the calibration curves, standard solutions were made using a commercial AFM1 standard solution (Sigma-Aldrich, Lawrence, MA, USA) with a concentration of 523 ng/mL. First, two intermediate standard solutions (5- and 50-fold dilutions in mobile phase) were made. Then, working standard solutions were prepared at concentrations of 0.02, 0.05, 0.1, 0.5, 1.0, 2.6, and 5.2 ng/mL. The standard and intermediate solutions were stored at −80 °C (±1 °C) in amber vials to prevent degradation. The linearity was validated in all chromatographic runs by examining the coefficient of determination (R^2^) of the calibration curves.

For methodological validation, the specificity was confirmed by the absence of interference from solvents or matrix components at the AFM1 retention time, verified through the injection of mobile phases, blank samples, naturally contaminated cheese samples, and standards. The linearity was excellent across the working range, with eight calibration curves prepared on different days yielding a mean coefficient of determination (R^2^) of 0.9995. The precision and recovery were evaluated using hard and fresh cheese samples spiked at four levels (50, 100, 250, and 500 ng/kg), with each analyzed in triplicate. The mean recoveries ranged from 87.8% to 92.6% in fresh cheese and 74.8% to 78.0% in hard cheese (see Supplementary Table S1). All results meet the requirements of Commission Implementing Regulation (EU) 2023/2782 [22]. The method achieved a limit of detection (LOD) for AFM1 of 8 ng/kg.

Furthermore, the accuracy of the results was externally validated through participation in the Progetto Trieste 2020 (CE1961/CM) international proficiency test. The laboratory’s performance was deemed satisfactory, achieving a z-score of 0.57, well within the acceptable range of −2 to 2. Regular recovery assays using cheese samples spiked at 100 ng/kg (AFM1) confirmed that the analytical method performed within acceptable requirements.

### 2.3. Estimation of Aflatoxin M1 Exposure Risk

The Margin of Exposure (MOE) approach was used to characterize the risk posed by cheese consumption, as recommended by the European Food Safety Authority [23]. An MOE that is lower than 10,000 is considered to indicate a potential health concern from exposure to AFM1 due to the genotoxic and carcinogenic nature of this mycotoxin. The MOE was calculated by comparing the estimated exposure to a dose-reference point as follows (Equation (1)):

(1) $$MOE=\frac{{BMDL}_{10}\;(ng/kg\;bw/day)}{EDI\;(ng/kg\;bw/day)}$$

BMDL_10_ represents the lower confidence limit of the benchmark dose associated with a 10% increase in cancer incidence in rodents. According to EFSA [23], the BMDL_10_ for AFM1 is a power factor of 0.1 derived from AFB1, which is 0.4 μg/kg bw/day. Equation (2) was used to calculate the estimated daily intake (EDI) of AFM1, based on the concentration of the contaminant in the cheese samples analyzed, the data on food consumption, and the average body weight (bw) of each population group.

(2) $$EDI\;(ng/kg\;bw/day)=\frac{mean\;AFM1\;(ng/kg)\times cheese\;consumption\;(kg/day)}{Average\;body\;weight\;(kg\;bw)}$$

For this deterministic approach, different food consumption scenarios were considered. In doing so, the mean AFM1 concentrations were calculated using the analytical results obtained for the cheese samples analyzed in this study.

Values below the limit of detection (LOD) were treated as half the detection limit (LOD/2). The cheese consumption data were obtained from the ENALIA survey published by the Spanish Agency for Food Safety and Nutrition [24]. This is an individual survey, conducted in accordance with a harmonized methodology agreed throughout Europe that provides precise information about the types and quantities of food consumed for assessing nutrient intake and for scientific research into exposure to chemical substances through food. The average consumption values and the 95th percentile (P95) were considered to characterize the average and high exposure (worst-case) scenarios, respectively. In addition, the EDI scenarios considered different age population groups as described in the food consumption database: toddlers (1–3 years), children (4–9 years), adolescents (10–17 years), and adults (18–74 years). Body weight assumptions were made according to EFSA reference values: 12 kg for toddlers (3 years), 23.1 kg for children (4–9 years), and 70 kg for adults (EFSA). For adolescents (10–17 years), an average body weight of 43.4 kg was applied, based on EFSA’s default for the 10–14-year age group, given the absence of harmonized data for older adolescents [25].

### 2.4. Data Analysis

A sample was positive for AFM1 when its concentration was above the limit of detection (LOD). Samples below the LOD were assigned a value of one half the LOD for the statistical analysis and exposure assessment purposes. The descriptive analysis of the mean, standard deviation (SD), and relative standard deviation (RSD%) was performed using Statistical Package SPSS v21 (IBM Corp, Armonk, NY, USA). First, the distribution of data was analyzed by the Kolmogorov–Smirnov and Shapiro–Wilk tests. Statistical differences were evaluated by means of the Mann–Whitney Test for the comparison between two groups. The Kruskal–Wallis Test was used for the comparison between more than two groups. Significant differences in the levels of mycotoxins were determined at a *p* value set to 0.05.

## 3. Results and Discussion

### 3.1. Occurrence of AFM1 in Commercial Cheese Samples

In this study, 100 commercial cheese samples from retail stores and supermarkets in northeast Spain were analyzed for AFM1. Overall, 51% of the samples exceeded the limit of detection, with concentrations ranging from 8.1 to 470.7 ng/kg. Data regarding AFM1 contamination in Spanish cheese remain scarce; notably, the prevalence observed here is significantly higher than that reported by Cano-Sancho et al., who detected no AFM1 in 72 composite samples analyzed [2]. Comparing these datasets reveals how the AFM1 risk profile in the Spanish market has evolved over the last decade, possibly due to changes in production practices and environmental conditions.

This increased prevalence must be viewed within the context of climate change. Rising temperatures and altered precipitation patterns in Europe are projected to increase the incidence of *Aspergillus flavus* in raw materials, particularly corn and corn silage used for dairy feed. This shift facilitates a higher carry-over of aflatoxin B1 from feed to aflatoxin M1 in milk and dairy products. Consequently, what was previously a negligible risk in certain European regions is emerging as a significant food safety challenge. It underscores the need for updated assessments such as the present study, which provides critical contemporary data capturing the real-world impact of the predicted European “mycotoxin shift”. Indeed, while previous meta-analyses are often based on older datasets, our findings (51% prevalence) align with recent environmental models [10,11,12] predicting an increase in aflatoxin incidence due to climate-related feed contamination, making this study a critical update for public health risk management.

Studies on this topic reveal considerable variability in AFM1 prevalence and contamination levels in cheeses. Much of this research focuses on assessing aflatoxin contamination in samples from the Middle East, where the climatic conditions and high dairy consumption increase vulnerability to aflatoxin contamination [26,27,28]. The available data indicate that countries in this region exhibit a pooled AFM1 prevalence in cheese of 63.6% [28]. Not only is the prevalence noteworthy, but the reported AFM1 concentrations have also been high in some cases. For example, levels ranging from 150 to 2410 ng/kg have been detected in feta cheese samples from Iran [29]. Similarly, studies from Turkey have reported AFM1 concentrations in cheese ranging from 15 to 3774 ng/kg [30,31,32]. In general, AFM1 contamination levels in these countries appear to be decreasing, though it still is necessary to implement stringent quality control measures throughout the dairy production chain to mitigate AFM1 contamination [33].

Significant research has also been conducted in countries where dairy product consumption is important and aflatoxins contamination is likely to occur, such as Brazil and Italy. In this context, Silva et al. [34] reported a high incidence of AFM1, detecting this mycotoxin in 100% of cheese samples (coalho and mozzarella) produced in the state of Pernambuco, in Brazil. The concentrations found ranged from 26 to 132 ng/kg. Corassin et al. [35], also in Brazil, found AFM1 in 47.4% of the analyzed samples, with concentrations between 17 and 695 ng/kg. Other Brazilian studies found a lower prevalence (30.5%), although the concentrations, ranging between 790 and 6700 ng/kg, were found to be very high [36].

In Italy, AFM1 was detected in over 83% of commercial cheese samples [37]. Most showed contamination levels between 50 and 150 ng/kg, while the remainder were generally in the range of 25 to 50 ng/kg, with only one sample exceeding 250 ng/kg.

A significant challenge in this field is the discrepancy in regulatory interpretation. Some authors consider concentrations above 50 ng/kg (maximum level for AFM1 in milk established by the European Union) significant [38], while others regard 250 ng/kg as the threshold for unacceptable AFM1 contamination in cheese [27,33] or even higher, depending on the regulations in their respective regions.

The references frequently highlight the lack of specific maximum level for AFM1 in cheese in the European Union. However, as stated in article 3 of Commission Regulation (EU) 2023/915 [15], when no specific maximum levels are established for processed foods, the concentration changes caused by processing must be factored in. Food business operators must provide and justify the specific processing factor for contaminant concentration during official controls. However, the lack of this information when analyzing commercial samples makes the evaluation of the results particularly challenging [39]. The Italian Ministry of Health defined provisional enrichment factors (EF), the ratio between the AFM1 concentration in cheese and that in milk, to assess the maximum acceptable AFM1 levels in these products: 5.5 for the hard cheese group (including semihard, hard, and very hard cheese categories) and 3.0 for the soft and semisoft cheeses [40]. Nevertheless, the limits derived from these enrichment factors are not cited in the literature as maximum reference limits.

In the present study, seven samples exceeded 50 ng/kg, though only one significantly surpassed 250 ng/kg. From these, only one of the samples was mature sheep’s cheese, while the remaining six samples were fresh cheeses made from cow’s milk. The generalizability of the results of the present pilot survey is further supported by the variety of cheese-making technologies examined. Since AFM1 has a high affinity for casein micelles, the concentration factor from milk to cheese varies, depending on the moisture content. By including a wide range of moisture profiles (from fresh to hard cheeses), our results provide a more comprehensive risk profile than studies focusing on a single cheese type.

AFM1 cheese contamination depends on many factors including the type of cheese, the technological strategies used during the manufacturing process, the amount of whey removed, the curdling temperature, the size of the grain after cutting the curd, the amount of fat, and the ripening time [3,18]. Clearly, the primary factor influencing cheese contamination is the concentration of AFM1 in the milk, which stems from the cattle’s feed.

Specifically, this study has found a significant difference (*p* < 0.05) in cheese contamination according to the dairy species. The concentration of AFM1 was higher in cheeses made from cow’s milk regarding the prevalence and contamination rate. Significant differences were also detected among cheeses as a function of the heat treatment applied to the milk. Consequently, AFM1 levels were found to be notably higher in the collected pasteurized milk cheeses compared with those produced from raw milk, a trend largely driven by specific high-concentration samples.

This study supports previous findings that also demonstrated a higher prevalence and concentration of AFM1 in cheeses made with cow’s milk compared to those made with sheep’s or goat’s milk [37,41,42]. For example, it was found that in cheese samples collected in Italy, the prevalence of AFM1 was higher in those from cow’s milk than in those from goat’s and sheep’s milk, although in this case, the differences were not significant [41].

The results obtained are consistent as well with the published data on milk contamination levels, which generally report lower concentrations in goat or sheep milk compared to cow milk [43,44]. These findings align with the lower rate of AFB1 to AFM1 transfer observed in these species. While a transfer rate ranging from 0.6% to 6% has been estimated in cows [5], lower values are generally reported for goats and sheep. In Assaf ewes, rates between 0.16% and 0.34% have been described [45], while in Lacaune ewes, the rate is approximately 0.54% [46]. Moreover, Mora-Medina et al. [47] reported extremely low rates around 0.06% for Florida dairy goats. Such differences may be explained by the distinct digestive systems and mechanisms of AFB1 assimilation among animal species. Factors such as the metabolic capacity, health status, and milk yield have also been shown to influence the transfer rate [45]. Additionally, variations in feeding practices may contribute to this disparity, as cattle feed tends to have a higher susceptibility to contamination with AFB1 [37]. While the labeling for all cheese samples indicated that the milk originated in Spain, the lack of farm-level traceability hinders the identification of the specific dairy herds or regions where the contamination occurred.

The type of heat treatment applied to the milk was another factor found to influence the AFM1 levels. As shown in Table 1, the AFM1 concentrations were significantly higher in cheeses made from heat-treated milk than in those produced from raw milk. This observation aligns with the existing literature, which frequently reports higher AFM1 levels in pasteurized or UHT products compared to raw milk [28,38]. For instance, Assem et al. found contamination rates of 73.6% in pasteurized milk versus 68.0% in raw milk [48]. Likewise, Ghaffarian-Bahraman et al. [49] observed significantly higher AFM1 concentrations in pasteurized milk samples (up to 24.89 ng/kg) than in raw ones (13.54 ng/kg). Notably, the observed AFM1 levels were higher in cheeses produced from pasteurized milk than in those made from raw milk; however, this difference was primarily driven by a single artisanal fresh cheese sample exhibiting an exceptionally high AFM1 concentration. Artisanal producers often source milk from a single farm, where a localized contamination event can result in a highly contaminated batch, whereas the industrial pooling of milk tends to dilute such toxins. Consequently, these findings should be interpreted with caution. Because AFM1 is known for its thermal stability, the observed differences likely stem from confounding factors such as milk sourcing or specific production systems rather than the pasteurization process itself. In this study, raw milk use was primarily associated with artisanal aged cheeses, which may involve different supply chain dynamics.

**Figure 1:**
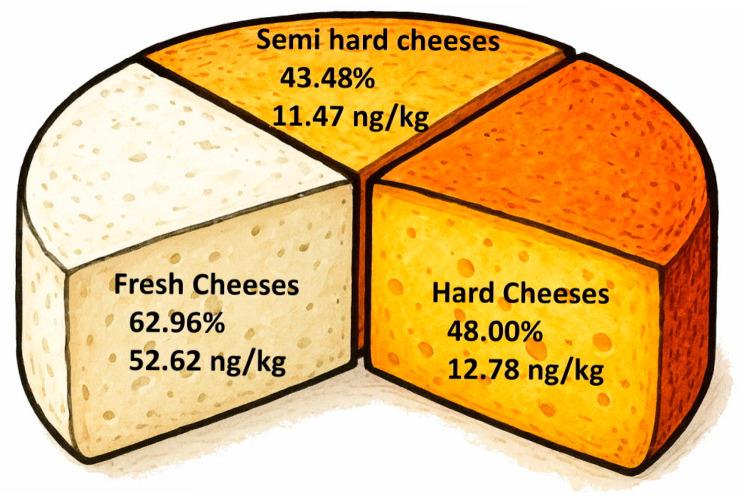
Distribution of AFM1 incidence (%) and concentration (ng/kg) in cheese samples according to the degree of ripening.

Regarding maturation, differences in AFM1 occurrence were observed based on the degree of ripening, though these did not reach statistical significance. Figure 1 shows that the prevalence (62.96%) and the average concentration of AFM1 in fresh cheese (52.62 ± 101.73 ng/kg) were higher than in aged cheese (48.0%; 12.78 ± 12.75 ng/kg) or semi-aged varieties (43.48%; 11.47 ± 9.55 ng/kg). Notably, six fresh cheese samples exceeded the 50 ng/kg threshold: two fell between 50 and 100 ng/kg, one between 100 and 200 ng/kg, and two between 200 and 250 ng/kg. Particularly noteworthy was one fresh cheese sample, which contained 470 ng/kg of AFM1, the highest concentration detected in this study.

AFM1 is highly heat resistant and remains stable throughout dairy processing; consequently, any contaminated milk will yield products that retain the mycotoxin [50]. During cheesemaking, AFM1 binds to the casein micelles, as the milk coagulates to form the curds. When whey containing much of the water and lactose is drained, the casein (and thus the bound AFM1) is concentrated into the cheese curd. Studies show that the AFM1 concentration in fresh curd can be 3 to 6 times higher than in the initial milk [51,52]. As a result, fresh cheese typically represents the peak concentration of AFM1 due to the initial enrichment factor from the milk’s casein. This is supported by numerous studies reporting higher prevalence and concentrations in fresh or white cheeses compared with aged varieties [13,27]. For instance, a comprehensive meta-analysis of Middle Eastern dairy products found a 95.7% pooled prevalence in fresh cheeses [28].

The concentration of AFM1 can further shift during the ripening process, though the results are often contradictory. On the one hand, moisture loss during extended ripening can further concentrate the toxin, leading some researchers to report higher AFM1 levels in aged cheeses [14,41]. On the other hand, microbial activity and physicochemical reactions may partially degrade or modify the AFM1 molecule [53]. Lactic acid bacteria and starter cultures have been shown to bind or degrade the toxin, potentially reducing its bioavailability. The final AFM1 level in ripened cheese likely reflects a dynamic balance between concentration via water loss and microbial detoxification. Therefore, discrepancies between different studies are not unexpected, as commercial samples often originate from diverse sources and are subjected to variable processing conditions. This variability highlights the importance of experimental studies designed to evaluate the distribution and behavior of contaminants during food production. As the observational design of the present study does not allow for causal conclusions regarding the processing effects, it is acknowledged as a limitation. While this work provides a valuable real-world snapshot of consumer exposure, controlled experimental studies using the same milk batch are required to quantify the exact impact of specific processing factors.

Inconsistencies in the reported effects of ripening and storage on AFM1 concentration and stability have been discussed in review articles by Campanolo et al. [3] and Sarmast et al. [14]. The latter specifically aimed to evaluate not only the distribution of AFM1 between curd/cheese and whey produced from contaminated milk but also the impact of the ripening and storage periods on AFM1 levels in the final product. These objectives were addressed through a meta-analysis. Sarmast et al. [14] concluded that although a modest but significant reduction in AFM1 levels (11%) occurs in cheese during ripening/storage, an effect that is more pronounced (21.1%) in samples made from milk containing starter culture, an increase of 77.2% in AFM1 levels was observed in cheese compared to the original milk after manufacturing.

This study examined the impact of the production method. Table 2 shows that artisanal cheeses exhibited slightly higher contamination levels than commercially produced ones. However, statistical analysis indicated that this difference was not significant (*p* > 0.05) and that the production method itself did not substantially contribute to the overall contamination.

Anfossi et al. [37] reported that Italian cheeses produced through artisanal methods were more highly contaminated with AFM1 than those produced in an industrial context. Industrial production typically involves “bulking”, the mixing of milk from diverse sources, which may dilute high AFM1 concentrations from a single farm. In contrast, artisanal producers often rely on a single milk source; if that specific source is contaminated, the resulting cheese will reflect high AFM1 levels. Furthermore, the modern technological processing and rigorous large-scale monitoring prevalent in developed regions contribute to lower AFM1 concentrations in industrial cheese. However, contrary to common belief, most reviewed studies found minimal differences between cheeses regarding their production system [36,52].

In summary, recent studies confirm that AFM1 remains a pervasive threat in the dairy chain, with global prevalence rates in cheese often exceeding 25–50% [54,55,56,57,58,59]. Furthermore, 2025 risk assessments in neighboring Mediterranean markets, such as Croatia, highlight that toddlers remain the most vulnerable group. In these regions, the estimated daily intakes (EDIs) are frequently found to approach or exceed the upper safety limits, underscoring the ongoing need for vigilant monitoring of the dairy market [60].

### 3.2. Assessment of the Risk of Exposure to AFM1

Milk and dairy products play an important role in the human diet across all age groups due to their high content of essential nutrients such as proteins, lipids, and calcium. However, the presence of AFM1 in these products poses a potential health risk to consumers. To evaluate whether the AFM1 levels in cheeses marketed in Spain represent a public health concern, this study used survey data to perform a risk assessment across various exposure scenarios. The resulting estimated daily intake (EDI) values are summarized in Table 3.

Overall, the EDI values calculated in this study were generally low, ranging from 0.004 to 0.081 ng/kg bw/day for average consumers and from 0.033 to 0.482 ng/kg bw/day for high consumers and tended to decrease in the older population groups. The exposure levels tended to decrease with age; this pattern is attributed to the higher food intake relative to body weight in infants and toddlers, as well as their higher consumption of fresh cheese, the most contaminated variety identified in this study, compared with ripened types.

While this study estimated low intake levels, significantly higher estimated daily intake (EDI) values, ranging from 10 to 100 times higher, have been frequently reported globally. Specifically, elevated EDI values associated with cheese consumption have been documented in Brazil, where values reached 0.040 ng/kg bw/day [61], with reports of 0.26 ng/kg bw/day for conventional cheese and 0.47 ng/kg bw/day for organic varieties [36]. In Iran, estimates ranged from 0.007 to 0.23 ng/kg bw/day across different demographics [27] and 0.03–0.05 ng/kg bw/day for traditional cheeses [62], and a meta-analysis reported 0.062, 0.071, and 0.144 ng/kg bw/day for men, women, and children, respectively [63]. In contrast, Greece reported a lower EDI of 0.01 ng/kg bw/day for feta cheese consumption [64].

The observed differences may be attributed not only to variations in AFM1 prevalence and concentration but also to differences in food consumption data, as dietary habits can vary significantly between populations and regions.

While the general population faces a low risk, these findings align with international research indicating that cheese consumption can pose a significant health threat to children. This risk is particularly acute in African and Asian regions, where higher contamination levels and increased consumption rates are prevalent [27,63,65].

Risk assessment using deterministic models yielded Margin of Exposure (MOE) values that were generally above the safety threshold of 10,000. Aligned with the exposure estimates, the risk calculations also identified children and toddlers facing the highest risk, though only toddlers with a high food consumption (p95) exhibited an MOE value below 10,000 indicating a potential health risk (Table 3).

Although the calculated MOE values suggest a low risk of exposure to AFM1, it is important to recognize that cheese usually is a minor contributor to the total AFM1 intake. This is particularly relevant for children, for whom milk remains the primary source of exposure; for instance, Udovicki et al. found that milk alone accounts for up to 52% of their total aflatoxin intake. [65]. In fact, the cumulative presence of AFM1 across the entire dairy chain remains a significant global public health concern [66].

Likewise, it should be noted that the exposure risk assessment parameter was calculated following the EFSA [23] scientific opinion, which states that, in the absence of a specific BMDL_10_ value for AFM1, the value of 0.4 μg/kg bw/day established for AFB1 should be used, applying an additional potency factor of 0.1, thus reflecting the lower toxicity of AFM1 compared with its precursor, AFB1. Consequently, MOE values derived from this approach are expected to be ten times lower than those calculated using the standard method. In this context, it is important to emphasize that just as more harmonized legislation regarding maximum AFM1 levels in dairy products is needed, a more consistent approach among authors when assessing the exposure risk to this mycotoxin would also be desirable.

In this study, a deterministic approach was used for exposure assessment, employing point estimates for AFM1 concentration and cheese consumption. While this method is widely accepted for preliminary risk characterization, it provides a conservative exposure estimate, particularly when the 95th percentile (P95) is used to represent high-level consumers. However, deterministic models do not account for the inherent variability and uncertainty in the data as effectively as probabilistic approaches [65,67]. For vulnerable population groups such as toddlers, using a probabilistic model helps us identify the specific details of those at the highest end of the exposure scale, rather than just looking at the average. Future research should aim to integrate these stochastic techniques to refine the risk characterization for mycotoxin exposure in the Spanish dairy chain.

## 4. Conclusions

While global meta-analyses have synthesized the broad risks of AFM1 in dairy, there is a lack of recent comprehensive data for Southern Europe, a region with high cheese diversity. This study fills this gap representing a market-based snapshot of AFM1 contamination in cheeses produced and commercialized in Spain in 2024. Our results demonstrate that despite historical improvements in farm hygiene and governmental oversight, AFM1 remains detectable in a substantial proportion of commercial cheeses in Spain; indeed, more than half of the analyzed samples contained quantifiable toxin residues. This persistence may be attributed to shifting climatic conditions that favor aflatoxin development.

While the AFM1 levels varied widely across products and were significantly associated with the milk source, the specific regional production patterns across the Spanish landscape have not been fully evaluated; consequently, regional variability in AFM1 occurrence cannot be entirely excluded. Notably, several samples exceeded 50 ng/kg, with some surpassing 250 ng/kg, concentrations that pose a significant concern for food safety and highlight a regulatory gap. While AFM1 is strictly regulated in raw milk, the lack of a dedicated statutory limit for finished cheese products complicates the enforcement of food safety standards across different processing methods.

In this study, cheeses produced from cow’s milk showed a higher prevalence and higher mean concentrations of AFM1 compared with those made from other milk types. Furthermore, while higher AFM1 levels were observed in pasteurized milk cheeses, this finding was largely driven by a limited number of highly contaminated artisanal samples rather than by the pasteurization process itself. Although the technological process did not exert a statistically significant influence on the final contamination, fresh cheeses exhibited higher contamination levels than semi-mature and mature cheeses, suggesting that the moisture content and maturation may modulate AFM1 concentration. Similarly, the comparison between artisanal and industrial manufacturing did not reveal significant differences, though the high variability within artisanal products suggests that a single contaminated milk source can significantly skew the mean values in smaller-scale production. Ultimately, these results represent an observational snapshot of the market and do not establish a causal link between processing methods and AFM1 levels. The type of milk used and its primary contamination level appear to be the determining factors. To definitively isolate the effects of specific processing variables from the initial milk contamination, further studies conducted under controlled experimental conditions using the same batch of raw milk are required.

Beyond providing updated occurrence data, this study contributes to the understanding of AFM1 exposure in a Southern European context under changing climatic conditions. The exposure estimates based on population consumption data in Spain indicate that AFM1 intake through cheese is very low for the general population. Nevertheless, toddlers with high consumption habits (95th percentile) exhibited Margin of Exposure (MOE) values below the benchmark threshold of 10,000, indicating a potential health concern for this subgroup.

The lack of harmonized regulatory frameworks, together with the heterogeneous risk-assessment approaches reported in the literature, makes the interpretation of results for these mycotoxins challenging. These findings highlight the need for continued monitoring of AFM1 in dairy supply chains and for targeted risk-management strategies that specifically address vulnerable population groups with higher dietary exposure.

## Figures and Tables

**Table 1:** Comparison of AFM1 occurrence according to milk origin characteristics.

Dairy Species/Milk Treatment	Positive Samples (%)	Mean ± SD (ng/kg)	Maximum (ng/kg)
Cow (*n* = 37)	67.57	43.43 ± 88.05	470.70
Sheep (*n* = 36)	38.89	11.41 ± 12.33	57.12
Goat (*n* = 21)	42.86	10.09 ± 8.77	34.38
Mixture (*n* = 6)	50.00	15.71 ± 14.01	34.75
Raw milk (*n* = 44)	38.64	10.73 ± 10.43	47.13
Pasteurized (*n* = 56)	60.71	33.06 ± 73.12	470.70

**Table 2:** Comparison of AFM1 occurrence according to the cheese production system.

Production System	Positive Samples (%)	Mean ± SD (ng/kg)	Maximum (ng/kg)
Artisanal (*n* = 53)	56.60	33.17 ± 75.10	470.70
Industrial (*n* = 47)	44.68	12.04 ± 12.00	57.12

**Table 3:** Estimated daily intakes (EDI as ng/kg bw/day) and Margin of Exposure (MOE) values for AFM1 from cheese consumption.

Age Groups	EDI (Average Consumers)	MOE (Average Consumers)	EDI (High Consumers)	MOE (High Consumers)
Toddlers	0.081	>100,000	0.482	8293
Children	0.025	>100,000	0.126	31,674
Adolescents	0.007	>100,000	0.055	72,619
Adults	0.004	49,193	0.033	>100,000

## Data Availability

The original contributions presented in the study are included in the article/Supplementary Material, further inquiries can be directed to the corresponding author.

## Supplementary Materials

The following supporting information can be downloaded at: https://www.mdpi.com/article/10.3390/foods15040720/s1, Figure S1: Geographical distribution of the 100 cheese samples across 12 Spanish autonomous communities. Red squares indicate the number of samples collected per region. The yellow-shaded areas on the map represent the northeastern regions of Aragón and Cataluña where the cheese samples were collected; Table S1: Method performance for aflatoxin M1 in spiked hard and fresh cheeses at different levels (3 replicates at each spiking level).
