# Supplementary material for: Assessment of Cheese Contamination and Its Contribution to Aflatoxin M1 Intake in the Spanish Population
Source: Foods. 2026 Feb 15;15(4):720. doi: 10.3390/foods15040720 (PMC12939574; doi:10.3390/foods15040720)
Supplement: Supplementary file 1 [file foods-15-00720-s001.zip › foods-4087604-supplementary.pdf]

Assessment of Cheese Contamination and its Contribution to Aflatoxin M1 Intake in the Spanish Population

Susana Lorán, Marta Herrera, Agustín Ariño and Teresa Juan

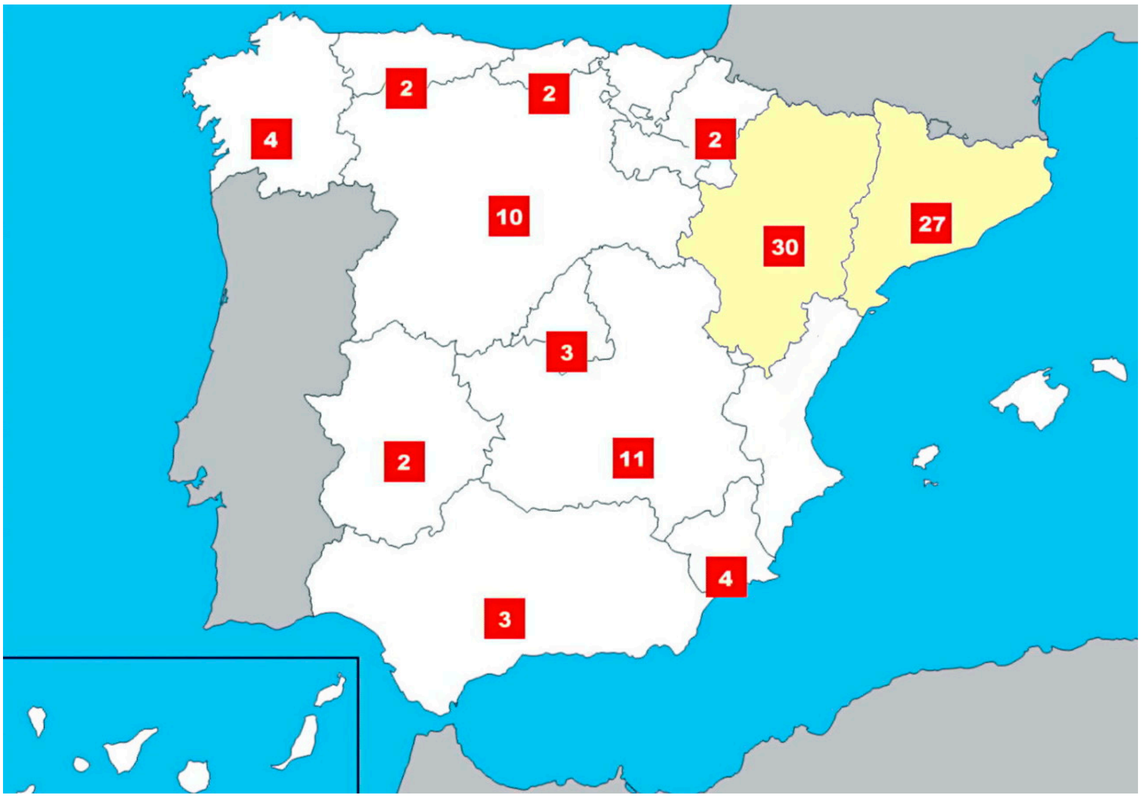

Figure S1. Geographical distribution of the 100 cheese samples across 12 Spanish autonomous communities. Created with d-maps.com and Microsoft Paint (Windows 10 version). Red squares indicate the number of samples collected per region. The yellow-shaded areas on the map represent the northeastern regions of Aragón and Cataluña where the cheese samples were collected.

Assessment of Cheese Contamination and its Contribution to Aflatoxin M1 Intake in the Spanish Population

Susana Lorán, Marta Herrera, Agustín Ariño and Teresa Juan

Table S1. Method performance for aflatoxin M1 in spiked hard and fresh cheeses at different levels (3 replicates at each spiking level).

Spiking level (ng/kg)	Cheese type	Recovery (%)	Repeatability (%RSD_r)	Reproducibility (%RSD_R)
50	Hard cheese	77.96	2.61	3.35
	Fresh cheese	92.60	5.87	6.33
100	Hard cheese	74.84	1.09	1.46
	Fresh cheese	98.11	3.69	5.54
250	Hard cheese	75.02	2.05	3.34
	Fresh cheese	91.85	3.90	4.39
500	Hard cheese	76.26	1.13	1.48
	Fresh cheese	87.83	2.70	2.75
